# White Matter Plasticity Keeps the Brain in Tune: Axons Conduct While Glia Wrap

**DOI:** 10.3389/fncel.2018.00428

**Published:** 2018-11-16

**Authors:** Zahraa Chorghay, Ragnhildur Thóra Káradóttir, Edward S. Ruthazer

**Affiliations:** ^1^Department of Neurology and Neurosurgery, Montreal Neurological Institute, McGill University, Montreal, QC, Canada; ^2^Department of Veterinary Medicine, Wellcome Trust - Medical Research Council Stem Cell Institute, University of Cambridge, Cambridge, United Kingdom

**Keywords:** activity-dependent, myelin, plasticity, oligodendrocyte, axon, conduction velocity, glutamate

## Abstract

Precise timing of neuronal inputs is crucial for brain circuit function and development, where it contributes critically to experience-dependent plasticity. Myelination therefore provides an important adaptation mechanism for vertebrate circuits. Despite its importance to circuit activity, the interplay between neuronal activity and myelination has yet to be fully elucidated. In recent years, significant attention has been devoted to uncovering and explaining the phenomenon of white matter (WM) plasticity. Here, we summarize some of the critical evidence for modulation of the WM by neuronal activity, ranging from human diffusion tensor imaging (DTI) studies to experiments in animal models. These experiments reveal activity-dependent changes in the differentiation and proliferation of the oligodendrocyte lineage, and in the critical properties of the myelin sheaths. We discuss the implications of such changes for synaptic function and plasticity, and present the underlying mechanisms of neuron–glia communication, with a focus on glutamatergic signaling and the axomyelinic synapse. Finally, we examine evidence that myelin plasticity may be subject to critical periods. Taken together, the present review aims to provide insights into myelination in the context of brain circuit formation and function, emphasizing the bidirectional interplay between neurons and myelinating glial cells to better inform future investigations of nervous system plasticity.

Myelin is the lipid-rich insulatory material that ensheathes axons in the central nervous system, increasing the speed of signal conduction and providing precise temporal control over the propagation of action potentials within brain networks. Despite the importance of myelination in circuit activity, relatively little is known about how myelination may itself be influenced by neuronal activity. Initial observations that myelination may be altered by changes in neural activity have spawned a growing interest in the phenomenon of myelin plasticity in recent years. This article will expand upon a number of excellent, recent reviews (e.g., Wang and Young, 2014; Fields, 2015; Purger et al., 2016; De Faria et al., 2017; Mount and Monje, 2017) to highlight how myelination may contribute to circuit function, to discuss some of the key evidence for activity-dependent changes in myelination, and to explore the underlying glia–neuron communication that makes this possible.

## Myelination Fine-Tunes Timing to Promote Circuit Functionality and Plasticity

**FIGURE 1:**
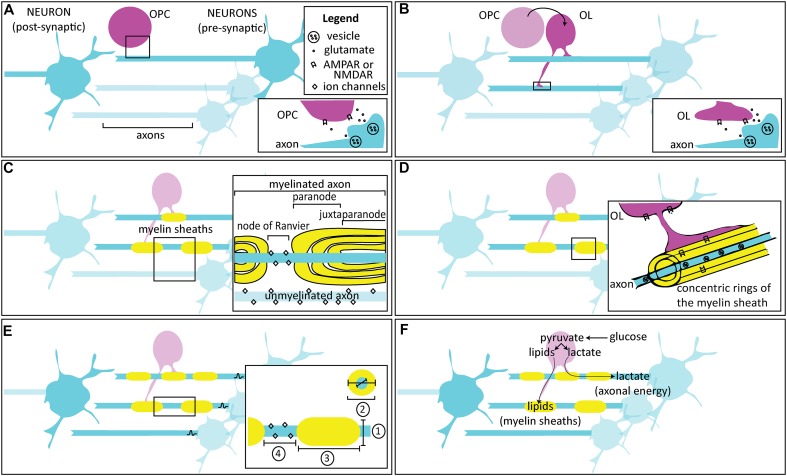
Activity-dependent myelination. **(A)** Oligodendrocyte progenitor / precursor cells (OPCs) are found in the developing circuit alongside neurons, and are thought to interact through glutamatergic signaling. Inset: Following an action potential, glutamate released from the axon is thought to bind to AMPARs and NMDARs on OPCs, either at a synapse or extrasynaptically. **(B)** OPCs differentiate into oligodendrocytes (OLs), which also interact with neurons in the developing circuit. Inset: Following an action potential, glutamate released from the axon is thought to bind to AMPARs and NMDARs on OLs, either at the axomyelinic synapse or extrasynaptically. **(C)** OLs enwrap concentric myelin sheaths around axons. One OL can enwrap multiple axons, while individual myelin sheaths along the same axon are wrapped by different OLs. The most important factor for myelin initiation is axon diameter, but neuronal activity may bias axon selection. Inset: In unmyelinated fibers, ion channels are distributed throughout the axon. Myelin ensheathment prevents current leakage by restricting ion flow primarily to particular, unmyelinated regions called the nodes of Ranvier. Immediately adjacent to the nodes are regions called paranodes, where the myelin loops contact the axons, and the regions further interior are called the juxtaparanodes. Nodes, paranodes, and juxtaparanodes are enriched with specific proteins to support their structural composition and function. **(D)** Over time, the concentric sheaths become compact myelin. In the adaptive myelination phase, changes to the extant myelin may occur in an activity-dependent manner. Inset: Glutamatergic receptors may be found within the sheathes themselves, where they are thought to respond to vesicular release and allow for myelinic calcium influx. **(E)** By varying the conduction velocity of the action potential, myelination can influence the arrival of axonal spikes, acting as a mechanism for regulating precise timing and synchrony in circuits. Mature, more myelinated circuits show inter- and intra-axonal variation in conduction velocity, depending on four factors (inset): 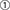 the axon diameter, 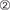 the myelin thickness, measured by g-ratio: a ratio of the inner to the outer diameter of the sheath, 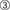 the length of continuous axonal segment that is wrapped, or the internode distance, and 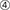 the node itself, including its geometry, and the composition and number of ion channels. **(F)** Other than plasticity, activity-dependent myelination is also thought to be important in maintaining circuits. Neuronal activity is detected by receptors in axons and cells of the OL lineage, which use their metabolites for maintenance of the myelin sheaths (e.g., through lipid synthesis) or for providing metabolic support (e.g., by shuttling lactate). This likely corresponds with a development switch in OLs from an earlier mode of myelin formation that utilizes mitochondrial-dependent aerobic respiration to a later, glycolysis-dependent mode that meets the energy demands for survival of the OL and of the associated axon especially during periods of increased activity.

Precise timing of the transmission of neuronal activity is critical for proper circuit function and also plays an important role in the experience-dependent plasticity that modulates brain wiring during development and throughout an organism’s lifetime. By preventing current leakage and reducing membrane capacitance, myelination of axons enables action potentials to depolarize more distant sites along the axonal membrane. Consequently, an action potential can rapidly propagate in a saltatory manner from one exposed gap in the myelin sheath, called a node of Ranvier, to the next, with ion flux restricted to the ion channels within the node (Figure 1C). Even if some nodes are inactivated, myelination can increase the length constant sufficiently to depolarize adjacent nodes (Tasaki, 1939). Greater internodal distances, along with more rapid membrane depolarization, allow for faster axonal conduction velocity. Thus, myelination of rapidly conducting axons enhances the speed and fidelity of information transmission. In any given axon, the speed at which an action potential travels is dependent upon a number of factors: (1) axon diameter, (2) myelin thickness, measured by g-ratio, which is ratio of the inner to the outer diameter of the sheath (Rushton, 1951; Waxman, 1975; Campbell et al., 2017), (3) length of continuous axonal segment that is wrapped (i.e., the internode distance) (Rushton, 1951; Brill et al., 1977), as well as (4) the nodal geometry itself (Arancibia-Cárcamo et al., 2017; Figure 1E). Interestingly, theoretical calculations based on measurements of these biophysical parameters have estimated that myelinated peripheral nerve is indeed optimized for maximum conduction velocity (Waxman, 1975). In contrast, the presence of diverse unmyelinated axons and myelinated axons that differ in these properties confers large heterogeneity in the conduction velocities in central nervous system circuits, implying a more elaborate role for central myelination in the temporal precision of functional circuits. Recent experimental evidence clearly indicates that rather than merely optimizing for high speed and fidelity of transmission along axonal tracts, myelination exerts a fundamental influence over the precise relative timing of signals, contributing to their integration into the network and even to their potential to drive changes in synaptic strength.

Within the early visual system, there are striking differences in conduction velocity along the optic nerve versus the optic tract demonstrating that axons are capable of locally regulating properties such as axon diameter (Baker and Stryker, 1990). In the thalamocortical projections of the somatosensory system, axons appear to regulate myelination to produce remarkably uniform latencies from thalamus to cortex despite a wide range of axonal distances to their different cortical target sites (Salami et al., 2003). Furthermore, these axons exhibit 10-fold differences in conduction velocity for their myelinated segments in the white matter (WM) compared to gray matter terminals where the same axons are not myelinated. This fine-tuning of signal propagation along axonal fibers allows information transmitted from wide-ranging locations across the brain to impinge onto higher order areas nearly simultaneously, permitting downstream neurons to integrate and process diverse inputs within millisecond timescales, unhindered by the different lengths of the individual axons themselves.

Precise timing in the transduction and transmission of sensory inputs is essential for accurate sound localization performed by circuits in the auditory hindbrain (Seidl, 2014). Because the ears are on opposite sides of the head, sound waves will arrive at each ear with interaural time differences (ITD) on the order of microseconds, depending on the relative proximity of each ear to the site from which the sound originated. Cochlear stimulation in response to the arrival of sound waves results in high fidelity activation of neurons in the cochlear nuclei associated with each ear. Bilaterally the relevant cochlear nucleus, called the nucleus magnocellularis in the avian auditory system, extends a bifurcating axon which innervates nucleus laminaris on both sides of the hindbrain. Nucleus laminaris is the first brain area that receives binaural inputs and it is organized such that relevant ITDs are represented along its width. This is accomplished by the implementation of a coincidence detector called a Jeffress model, in which inputs from the two cochlear nuclei enter opposite sides of nucleus laminaris and the coincident arrival of action potentials representing the two ears is detected by the postsynaptic neurons (Jeffress, 1948; Sullivan and Konishi, 1986; Overholt et al., 1992). What is truly remarkable here is that the much greater lengths that must be traversed by axons from the contralateral side of the brain would effectively cause all those inputs to arrive too late to coincide with ipsilateral signals, yet because of dramatic differences in myelin internodal lengths and relative axon diameters of the ipsilateral and contralateral branches of the bifurcating magnocellularis axons, coincident arrival with microsecond accuracy is achieved (Seidl et al., 2010). Fine-tuning of the relative speeds of signal propagation can also be found in the mammalian auditory system (Ford et al., 2015), where it has been shown that sensory deprivation through ear-plugging results in significant reductions in myelin sheath thickness (Sinclair et al., 2017). Thus, auditory brainstem fibers exploit differences in myelination, including gradations in nodal diameter, internodal length, and myelin thickness, to optimize their relative speed and synchronization.

**FIGURE 2:**
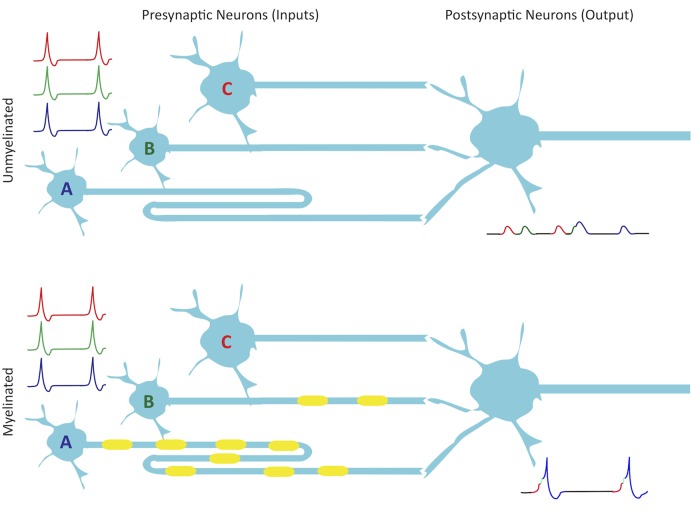
Myelination improves synchrony. Presynaptic neurons A, B, and C provide input to their postsynaptic partner. Although they fire simultaneously, A has a longer axon length than B, which is longer than C. All three presynaptic axons have the same diameter so if they are unmyelinated, they have a constant conduction velocity, which results in non-synchronous activation of excitatory postsynaptic potentials due to conduction delays along the axons. Myelination could modulate conduction velocities in such a way as to allow for synchronous spike arrival at synapses. With spikes from the myelinated inputs arriving coincidentally, summation in the postsynaptic neuron reaches threshold on multiple occasions, leading to a number of action potentials. Small shifts in arrival time could also modulate STDP of inputs.

These studies support the notion that the control of myelination along and between axons might be a strategy for modulating conduction velocity to regulate the functional integration of inputs into neuronal circuits (Figure 2). Both in development and in adulthood, plasticity mechanisms also rely critically on precise timing (Sjöström and Gerstner, 2010). Hebb (1949) postulated that synapses could be strengthened when a presynaptic neuron “repeatedly or persistently” participates in exciting its postsynaptic partner to fire action potentials, implying temporal correlation of inputs. Conversely, synapses might be expected to weaken when a presynaptic neuron consistently fails to help drive its postsynaptic partner (Stent, 1973). These Hebbian rules of plasticity constitute cellular level manifestations of the cooperativity and competition that guide the development, refinement, and maintenance of circuits at the system level. They underlie the activity-dependent self-organization of developing circuits, which calls for the selection of an increasingly restrictive subset of inputs as functional maps refine over time.

The precise timing of the asymmetry between the arrival of presynaptic spikes and the firing of postsynaptic cells is the basis of a type of Hebbian learning called spike-timing-dependent plasticity (STDP; for a comprehensive overview, see the e-book by Markram et al., 2012). STDP is defined by timing windows within which a presynaptic cell that fires *before* its postsynaptic partner undergoes synaptic strengthening, whereas activation of the presynaptic cell *after* its postsynaptic partner weakens the synapse. The specific direction of the synaptic modification may differ between brain areas, but in most cases the timing window to induce the change lasts only a few tens of milliseconds (Dan and Poo, 2004). The first *in vivo* demonstration of STDP was performed in the developing retinotectal system of the *Xenopus laevis* tadpole (Zhang et al., 1998). In the *Xenopus* visual system, STDP has since been implicated in activity-dependent motion selectivity (Engert et al., 2002; Mu and Poo, 2006), receptive field refinement (Tao and Poo, 2005; Vislay-Meltzer et al., 2006; Dong et al., 2009), and recurrent excitation (Pratt et al., 2008). Across different systems, including mammalian somatosensory (Feldman, 2000) and visual (Yao and Dan, 2001; Sjöström et al., 2003) cortices, STDP is an important contributor to the activity-dependent development and refinement of sensory computations (Richards et al., 2010), particularly in circuits that process fast timescales and correlations (Butts and Kanold, 2010). Importantly, it has been observed that the activity requirements for STDP vary considerably across brain regions, among synapse types, and even within the same cell at different dendrites, and this variability is thought to be important for coordinating circuit development (reviewed by Caporale and Dan, 2008; Sjöström and Gerstner, 2010). For example, at the same spatial locations, over the course of development, the spatiotemporal window for neighboring inputs to cooperatively elicit plastic changes at synapses becomes increasingly narrow, conducive to progressively exacting fine-tuning of the circuit (Tao et al., 2001; Takahashi et al., 2012). Timing is integral to plasticity, and therefore mechanisms that control the timing of impulses will influence plasticity.

A study by Yamazaki et al. (2007) recorded from myelinating cells, the oligodendrocytes, in the alveus of rat hippocampal CA1, and showed that they exhibited membrane depolarization in response to high frequency and theta burst stimulation of neuronal fibers, mediated by glutamate receptor activation. Remarkably, they found that depolarization of oligodendrocytes could shorten conduction velocities of CA1 pyramidal cell axons by nearly 10% within a matter of seconds. Such changes in axonal conduction velocity, in conjunction with STDP mechanisms, could result in dramatic changes in synaptic strengths, potentially even converting a synapse that had been undergoing synaptic depression to one that strengthened instead. Moreover, even small changes in conduction velocity can have profound effects on oscillatory network activity, converting synchronous inputs into sources of destructive interference that can disrupt the entrainment of network oscillations (Pajevic et al., 2014), such as the theta-gamma coupling in the hippocampus that has been shown to be essential for effective learning and memory recall (Tort et al., 2009). Examples of how changes in myelin form or function may regulate conduction velocity to affect synaptic plasticity are still largely absent from the literature, but this rather unconventional hypothesis that dynamic control of conduction velocity may function as a potent form of metaplasticity is gaining traction.

## Experience and Training Induce WM Changes

Myelin changes may be reflected at the gross level as WM changes, which have been extensively observed in the human brain using diffusion tensor imaging (DTI) to measure fractional anisotropy (FA) (reviewed by Zatorre et al., 2012; Fields, 2015; Sampaio-Baptista and Johansen-Berg, 2017). FA is a measure of the directional dependence of water diffusion, ranging from isotropic (free) diffusion to diffusion confined to one direction. It is based on the assumption that axonal and myelin membranes within fiber tracts constrain the normally random diffusion of water molecules to instead diffuse anisotropically along the lengths of the fibers. It has therefore been widely used to reveal differences in anatomical characteristics, such as axon dimensions, fiber density, and myelination (Beaulieu, 2002). To date, WM changes have been observed to occur during development, adulthood, and aging. Aging in particular has been linked to changes in WM volume (reduction), WM lesions as seen by hyperintensities, and WM microstructure as measured by FA decreases, with social and cognitive activities acting in a neuroprotective fashion to decrease the magnitude of these changes (meta-analysis by Anatürk et al., 2018), hinting that WM neuroanatomy may be altered by experience over the lifetime of an organism.

Cross-sectional studies comparing experts to novices have revealed FA increases in WM tracts connecting brain regions previously identified as the neuronal correlate of the cognitive domains being studied. Cognitive studies of WM have shown FA increases related to memory, reading ability, grammar learning, and mental rotation (Sampaio-Baptista and Johansen-Berg, 2017). FA increases have been particularly well-characterized in musicians, making the case for musical training as a model of studying plasticity, and especially WM plasticity, with neuroimaging (Herholz and Zatorre, 2012). As a specific example, consider how piano practice in different stages of life has been associated with FA increases in WM. In childhood, FA increases are seen in the following WM regions: (1) the internal capsule carrying corticospinal tracts from the primary sensorimotor and premotor cortices; (2) the superior and inferior frontal regions, including areas of the premotor cortices needed for bimanual coordination and movement sequences; and (3) the corpus callosum connecting auditory regions of the cortex and superior frontal regions (Bengtsson et al., 2005), especially its isthmus in musicians who begin training prior to 7 years of age (Steele et al., 2013). In addition to the FA increases in parts of the internal capsule, piano practicing in adulthood but not in childhood leads to FA increases in fiber bundles of the arcuate fasciculus connecting the temporal and frontal lobes (Bengtsson et al., 2005). However, cross-sectional studies have a caveat: even though they may attempt to reduce confounds by matching participants, such as, for culture when examining reading skills (Carreiras et al., 2009) or for years of training and experience in musicians (Steele et al., 2013), by design, they cannot parse out pre-existing from experience-dependent structural changes.

This disadvantage can be reduced using a longitudinal approach to examine activity-dependent WM changes, since structural images acquired before and after task-specific training can be compared. In one of the first longitudinal studies of myelin plasticity, Scholz et al. (2009) showed that 6 weeks of juggling training led to a FA increase in WM underlying the right posterior intraparietal sulcus. This sulcus has been well characterized in its role in integrating sensorimotor information for the guidance of motor behavior, such as to mediate reaching and grasping behavior in macaques (Borra and Luppino, 2016). The positive correlation between training and FA was, similarly, observed for working memory training, including FA increases in WM regions adjacent to the intraparietal sulcus, around the body of the corpus callosum and adjacent frontal lobe, and near the border between the frontal and parietal lobes that have been previously implicated in working memory (Takeuchi et al., 2010). Using FA as a readout of myelination in human neuroimaging has been validated with animal studies that also observed changes in a molecular marker of myelin called myelin basic protein (MBP): rats showed increases in both FA and MBP expression, as compared to controls, in the corpus callosum after 5 days of training on the Morris water maze (Blumenfeld-Katzir et al., 2011), and in the motor cortex WM tracts contralateral to the limb trained to grasp a food pellet reward (Sampaio-Baptista et al., 2013). The link between task demands and FA increases in corresponding brain regions tends to be correlated with the amount of training rather than outcome (final skill level), perhaps pointing to different anatomical correlates of learning versus performance (Scholz et al., 2009; Takeuchi et al., 2010). Additionally, in these studies, gray versus WM structural changes seem to be relatively independent, both spatially and temporally. These observations suggest that learning (training) but not doing (performing a learned task) involves strengthening of functional connectivity, actively recruiting myelination mechanisms.

However, experience does not always induce FA increases. While most research in musicians reported FA increases, studies in dance and other whole-body motor activities showed decreased FA in WM tracts like the corticospinal tract, superior longitudinal fasciculus, and corpus callosum (Giacosa et al., 2016). Improvement on a car-racing game in humans (and on the Morris water maze in 4 months old rats) led to FA decreases in the fornix (Hofstetter et al., 2013), a WM structure that carries efferents from the hippocampus, a structure implicated in spatial learning and other tasks. FA decreases in WM connectivity of motor and association cortical areas were also observed in a whole-body balancing task: FA was negatively correlated with learning the task and performance improvement in the left lateral prefrontal cortex (PFC) and right motor cortex, and with muscular imbalances in the left superior parietal and right occipital WM and in the left cingulum (Taubert et al., 2010). The authors suggested that these inverse FA changes may be due to the proportion of crossing versus non-crossing fibers that are recruited in a task. FA is a direction-dependent measure, so its readouts regarding WM changes can be confounded by whether local versus distributed networks were recruited. Imaging whole mouse brains for FA changes as well as MBP immunolabeling showed a correlation between the two measures in WM regions of coherent fiber orientations and low fiber dispersion, but not in areas of crossing or complex fiber architecture (Chang et al., 2017). Therefore, some studies show FA decreases rather than increases because FA values are modulated but not necessarily dictated by myelination (Beaulieu, 2002), also reflecting myelin-independent factors like fiber organization (especially crossing fibers), astrocyte number and activity, and angiogenesis (Zatorre et al., 2012; Sampaio-Baptista and Johansen-Berg, 2017). Taken together, research over the last decade-or-so successfully challenges our traditional notions of experience-dependent changes in brain circuits, by showing that not just synapses but also myelin and WM can be altered by experience, and therefore, deserve attention in studies of plasticity. Examining WM with FA and other neuroimaging measures has its caveats requiring careful consideration and interpretation, but nonetheless provide us with insight into human functional neuroanatomy and plasticity. Continued investigations with neuroimaging should be complemented where possible with reductionist cellular and molecular approaches to further enhance our understanding of experience-dependent modulation of WM connectivity.

## Neuronal Activity is Associated with Myelination Changes at the Cellular and Molecular Levels

### Cellular Markers of Oligodendrocyte Differentiation and Myelination

Myelin is produced and ensheathed around axons by glial cells called oligodendrocytes (OL). The OL lineage arises from embryonic neural progenitors, which give rise to OL precursor or progenitor cells (OPC), finally differentiating into OLs (Figures 1A,B). Single-cell transcriptomics show that although early progenitors of the OL lineage converge in terms of their transcriptional identities, OL heterogeneity can nonetheless be observed at later stages (Marques et al., 2016; Zeisel et al., 2018). This suggests that OL diversity arises from secondary diversification rather than developmental patterning, hinting at the role of the local environment in maturation of the OL lineage.

The unique expression profiles of the OL lineage provide useful molecular markers for studying myelination (see reviews by Mitew et al., 2014; Ackerman and Monk, 2016). For example, expression of the transcription factors Sox10, Olig1, and Olig2 turns on as OPCs first appear and stays on as they further develop and differentiate. OPCs are often identified distinctly from OLs by the proteoglycan neuron-glial antigen 2 (NG2) or platelet-derived growth factor α receptor (PDGFαR), and earlier studies also use the expression of O-2A for OPCs though it is slightly less restricted to the OL lineage. These markers can be used to determine the number and proliferation of OPCs, and to study oligondendrogenesis. An antibody commonly used to label OL cell bodies is CC1, which detects anti-adenomatous polyposis coli (APC) (Bin et al., 2016). Pre-myelinating and myelinating OLs express proteolipid protein (PLP), and the enzyme 2′,3′-cyclic-nucleotide 3′-phosphodiesterase (CNP). Only myelinating OLs express myelin associated glycoprotein (MAG) and MBP, enriched in their ensheathing myelin processes. There is a wide array of antigens beyond those noted here, and we will mention these as necessary throughout this review.

### Myelin Levels and Morphology Are Influenced by Neuronal Activity

The process of myelination is thought to be subject to both activity-independent and activity-dependent influences. The most prominent cue for myelin initiation is considered to be axon diameter, since even engineered nanofibers can become ensheathed with a physiologically appropriate sheath length reflecting the regional identity of the OL – cortical OLs produce shorter ensheathed segments than spinal cord OLs (Bechler et al., 2015). Other cues intrinsic to heterogeneous populations of OPCs and OLs are more difficult to parse out, but may nonetheless underlie the variation in myelin initiation, with neuronal activity and experience fine-tuning this myelination later on (see review by Bechler et al., 2018), akin to guidance cues establishing the initial coarse neuronal circuit which then undergoes activity-dependent refinement.

The idea that changes in neural activity may alter myelin itself is not new. One of the first reports of the effect on myelination of visual experience came from experiments involving dark-rearing of mice, which delayed differentiation of their optic nerve fibers. Mice reared in complete darkness until postnatal day (P) 20 had a reduced number of myelinated fibers compared to controls, especially in the number of coarser, heavily-myelinated fibers, which were reduced by almost 60% (Gyllensten and Malmfors, 1963). In transgenic mice engineered to express green fluorescent protein in myelin, monocular deprivation from P15 to P32 resulted in a higher proportion of short myelin internodes and slower conduction velocity in the deprived ON, compared to the non-deprived control optic nerve (Etxeberria et al., 2016). Conversely, artificial early eye opening in rabbits at P5 accelerated optic nerve myelination between P7 and P10, compared to controls with normal eye opening, although the myelination reached equal levels in both groups by 3 weeks (Tauber et al., 1980). The change in myelination was observed as increased levels of MBP and PLP, and as increased activity of CNP. Numerous other deprivation experiments have produced a wide range of outcomes depending critically on the exact conditions and brain areas studied (reviewed in De Faria et al., 2017).

In addition to consequences on visual processing, myelin-related changes may also underlie more complex behaviors. MBP expression levels change in the corpus callosum following Morris water maze (Blumenfeld-Katzir et al., 2011) and motor (Sampaio-Baptista et al., 2013) training. Mice that were socially isolated from P21 showed changes in myelination analyzed in the medial PFC at P65, which correlated with deficits in PFC-dependent behavior at P50, including in social interaction and working memory tasks (Makinodan et al., 2012). Specifically, in the medial PFC of isolated mice, OLs had shorter, less-branched processes, fewer internodes per cell, reduced mRNA levels of MBP and MAG, and reduced myelin thickness as assessed by electron microscopy.

Experimentally manipulating electrical activity and vesicular release in neurons also modulates myelination. In dissociated embryonic mouse cultures, administration of the voltage-gated Na^+^ channel blocker, tetrodotoxin (TTX), during a period immediately prior to the onset of myelination, reduced the number of myelinated segments (Demerens et al., 1996). Conversely, alpha-scorpion toxin, a drug that slows Na^+^ channel inactivation, increased the number of myelinated segments. In dorsal root ganglion neurons co-cultured with OPCs, blocking synaptic vesicle exocytosis by pre-treating the neurons with botulinum toxin or tetanus toxin led to fewer myelin segments, lower MBP expression, and restricted MBP mobility that was visualized by tagging MBP with photoactivatable green fluorescent protein (Wake et al., 2011). A number of studies have investigated activity-dependent myelination in the spinal cord of the zebrafish *(Danio rerio)*, an ideal model system for *in vivo* studies, amenable to genetic and pharmacological manipulation together with live imaging. Neurons expressing tetanus toxin light chain to impair vesicular release were less likely to be selected for myelination, and those that were myelinated had reduced sheath lengths compared to controls (Hines et al., 2015; Mensch et al., 2015). Competition between neurons may bias axon selection for myelination: Tetanus toxin-expressing neurons exhibited sheath lengths comparable to untransfected axons when treated with TTX to prevent competition (Hines et al., 2015). Treatment with γ-amino-butyric acid (GABA)-A receptor antagonist, pentylenetetrazole, increased myelin sheath number, presumably by elevating neural activity, as the increase was prevented by tetanus toxin expression (Mensch et al., 2015). In subsequent work, although the impairment in myelination of tetanus toxin-expressing axons was robust for reticulospinal neurons, there was no effect on commissural primary ascending neurons, highlighting that myelin plasticity differs from tract to tract (Koudelka et al., 2016). Elucidating activity-dependent mechanisms underlying axon selection for myelination will be an important direction for future investigations.

### Myelinating Glia Number Is Regulated by Neuronal Activity

Other than direct changes to the extant myelin sheaths, an important way that myelin plasticity may be regulated is through the genesis, differentiation, and proliferation of myelinating glia. In the rat visual system, in optic nerve transected at P8, blocking action potentials of retinal ganglion cells (RGCs) with TTX led to a reduced number of mitotic OPCs (Barres and Raff, 1993). Unlike in the optic nerve, TTX administration to cerebellar slice cultures had the opposite effect of increasing OPC proliferation and differentiation into OLs (Fannon et al., 2015). In a toxin-induced focal demyelination lesion of the caudal cerebellar peduncle, TTX treatment increased OPC proliferation but impaired remyelination, indicating differentiation of OPC that failed to properly myelinate axons (Gautier et al., 2015). The decreases in OPC and OL numbers following axonal activity blockade are transient with OL numbers recovering by a later time point (Barres and Raff, 1993; Demerens et al., 1996). In the corticospinal tract, 10 days of daily unilateral stimulation with an implanted electrode increased OPC differentiation and proliferation (Li et al., 2010). Optogenetic stimulation of layer V projection neurons in the mouse premotor cortex promoted OPC proliferation, oligodendrogenesis, and increased myelin sheath thickness (Gibson et al., 2014). This increase in oligodendrogenesis and myelin sheath thickness was also observed in cortical pyramidal neurons, which were stimulated by administering clozapine-*N*-oxide to activate hM3Dq DREADDs (designer receptor exclusively activated by designer drug) electroporated *in utero* in mice (Mitew et al., 2018). Of note, in this study, immunogold labeling of the green fluorescent protein that was co-expressed with the hM3Dq DREADDs showed that the increase in myelin thickness was preferential to stimulated rather than unstimulated axons.

Physiological sensory experience can also modulate myelinating glia number. Whisker clipping decreased the density of CC1+ OLs in the deprived somatosensory cortex, likely through reduced survival or increased apoptosis of NG2+ OPCs that were differentiating into CC1+ OLs (Hill et al., 2014). The barrel cortex is organized such that CNP+ NG2+ cell density is higher in the barrel septa than in the barrel cores where thalamocortical afferents terminate. However, following removal and cauterization of whiskers in mice, which induces reorganization of inputs to the corresponding barrel cortex, NG2+ cell proliferation becomes more uniform across the septa and cores (Mangin et al., 2012). In the same study, dark-rearing was shown not to alter NG2+ cell distribution and density in the primary visual cortex. In contrast, monocular deprivation decreased OPCs and increased mature OLs in the corresponding optic nerve and optic tract (Etxeberria et al., 2016). These studies reveal that the effects of sensory deprivation on OPCs and OLs vary depending on the type of sensory deprivation, modality, and specific brain region.

Physical exercise has also been shown to increase differentiation of NG2+ OPCs into mature OLs and decrease cell proliferation (Simon et al., 2011). When mice were trained to run on a complex wheel, which has irregularly-spaced rungs, learning this task coincided with increased OPC proliferation and OL production in the corpus callosum (McKenzie et al., 2014). This study was particularly convincing regarding the involvement of glia in motor learning: preventing oligodendrogenesis after complex wheel training, using a tamoxifen-inducible Cre deletion of the myelin regulating factor (Myrf), did not lead to the learning impairments seen when oligodendrogenesis was prevented prior to training. Using *in situ* hybridization of a new molecular marker identified to be preferentially upregulated in early differentiating OLs, Enpp6, a choline-specific ectonucleotide pyrophosphatase/phosphodiesterase, learning on the complex wheel task was shown to induce new OL differentiation in the subcortical WM within just 2.5 h and at 4 h in the motor cortex (Xiao et al., 2016). Differences in the generation of new OLs increased further over 24 h, and were seen even 8 days later, suggesting sustained differences in OL number during motor learning. Environmental enrichment also affects the populations of OLs, increasing proliferation of primarily Olig2+ cells in the amygdala (Okuda et al., 2009) and the number of CNP+ cells counted in the corpus callosum (Zhao et al., 2011). Future research should investigate the bases for the differences in responses of myelinating glia depending upon the brain region, developmental age of the animal, and type of experimental paradigm.

## Neuron-Glial Signaling for Myelin Plasticity

Since myelination can be influenced by neuronal activity, it follows that neurons and myelinating glia must somehow communicate with each other. It was previously suggested that axonal activity leading to the accumulation of signaling molecules and ions in the extracellular space could be sensed by glia (Ransom and Orkand, 1996). A number of studies show that OPCs and perhaps OLs express various glutamatergic receptors, including α-amino-3-hydroxy-5-methyl-4-isoxazolepropionic acid receptors (AMPAR) and N-methyl-D-aspartate receptors (NMDAR) (Figures 1A,B). Metabotropic glutamate receptors and other neurotransmitter receptors may also be involved (see review by Bakiri et al., 2009). For example, currents recorded in hippocampal OPCs include both a fast AMPAR-mediated component, and a slower component, mediated by GABA (Lin and Bergles, 2004). Purinergic receptors participate in myelination: in dorsal root ganglion and OPC cocultures, the inhibition of OPC proliferation, and the promotion of differentiation and myelination in response to action potential firing is prevented by blocking adenosine receptors, and adenosine application mimics the effect of action potentials (Stevens et al., 2002). NG2+ OPCs express voltage-gated ion channels, including for Na^+^, K^+^, Cl^-^, and Ca^2+^ ions, hyperpolarization-activated cyclic nucleotide-gated (HCN) channels, and transient receptor potential (TRP) channels (see review by Larson et al., 2016). Electrical activity could also directly stimulate the release of factors or exosomes from neurons or glia (see review by Wang and Young, 2014). These factors may include platelet-derived growth factor, basic fibroblast growth factor, and other pro-myelinating factors. Exosomes may contain proteins important for myelination, as well as microRNAs that act as epigenetic regulators of OPC differentiation. While previous reviews have examined the evidence for glutamatergic receptors on myelinating glia (Káradóttir and Attwell, 2007; Bakiri et al., 2009; Spitzer et al., 2016), here, we focus on ionotropic glutamatergic transmission as a mechanism of myelin plasticity.

The “axomyelinic synapse” (Stys, 2011) has been suggested as a potential cellular structure where important neuron-glia signaling would occur (Figure 1B). This model requires that myelinating glia express neurotransmitter receptors, form synapses with neurons, and exhibit electrical responses. Morphologically defined synaptic junctions between unmyelinated axons and OPCs in the rat corpus callosum were observed by immunofluorescence and electron microscopy (Kukley et al., 2007; Ziskin et al., 2007). They appeared to be the sites of neurotransmitter release from unmyelinated axons onto Ca^2+^-permeable AMPARs on postsynaptic-like densities of OPCs, However, investigations in DRG and OPC cocultures implicated glutamate and ATP release directly from axon varicosities to nearby OPC processes at axoglial contact sites, which lacked the characteristic features of synapses and likely arose from spontaneous vesicular release (Wake et al., 2015). This suggests that axonal exocytosis promoting myelination is not necessarily restricted to a synaptic structure, although some alternative explanations for these contradictory observations may include differences in cerebral versus spinal cord neuron–glia communication, timing of observation at day 1 upon addition of OPCs rather than waiting for 12 h or more for synapses to form, as well as potential artifacts of the coculture system.

Crucial to understanding the neuron-glial signaling that underlies myelin plasticity are observations of electrical responses in cells of the OL lineage, including excitatory postsynaptic currents (EPSCs) elicited by glutamate, and even action potentials. Action potentials were observed in a subset of NG2+ OPCs in cerebellar slices expressing voltage-gated Na^+^ and K^+^ channels (Káradóttir et al., 2008), and in a subset of pre-myelinating OLs in the auditory brainstem following glutamate stimulation (Berret et al., 2017). These action potentials are then thought to trigger Ca^2+^ influx in OLs and myelin sheaths. Following an induction of vesicular glutamate release in the optic nerve by electrical stimulation or sucrose application, *ex vivo* Ca^2+^ imaging showed Ca^2+^ influx from axonal stores into the myelin (Micu et al., 2016). In hindbrain neurons projecting to the zebrafish spinal cord, electrical stimulation increased Ca^2+^ in OL processes and sheaths, and led to sheath elongation, while TTX injection had the opposite effect (Krasnow et al., 2018). Interestingly, calcium imaging of GCaMP6s-expressing OLs revealed Ca^2+^ signatures for sheath retraction versus extension: high-amplitude, long-duration Ca^2+^ transients precede sheath retractions, while higher- frequency transients that are smaller and shorter are associated with sheath elongation (Baraban et al., 2018; Krasnow et al., 2018).

### Roles of AMPARs on Myelinating Glia

AMPARs have been observed on OPCs (see reviews by Káradóttir and Attwell, 2007; Bakiri et al., 2009). Pharmacological AMPAR activation decreases OPC proliferation and differentiation in culture (Gallo et al., 1996), on cells isolated from cerebellar slices (Yuan et al., 1998), and in cerebellar slices *in situ* (Fannon et al., 2015). Correspondingly, administration of TTX and the AMPAR non-competitive antagonist GYKI, also reduced MBP expression and the elongation and branching of OPC processes, thus decreasing overall myelination (Fannon et al., 2015). In mouse subcortical WM, knocking out AMPAR subunits increased the number of apoptotic OLs at P14, and consequently, reduced the number of myelin sheaths, but did not appear to affect OPC differentiation and proliferation, or other properties of myelin, such as sheath length (i.e., internode length of the axon) or thickness (Kougioumtzidou et al., 2017). The seemingly contradictory effects of decreased OPC proliferation and differentiation, and myelination, following AMPAR activation, but no effect with AMPAR knockout, may be reconciled in light of the limitations to current approaches. First, AMPAR agonists and antagonists may work on cells beyond the intended targets, especially with prolonged drug exposure. On the other hand, with a genetic approach, most knockout studies affect AMPARs from embryogenesis instead of at a developmentally relevant timepoint, allowing for significant alterations in the signaling cascades or in the microenvironment, which together prevent an accurate recapitulation of the baseline, physiological condition. Better insight into the complexity of AMPAR-mediated effects requires the continued development and utilization of experimental manipulations that are more temporally and spatially selective, involving inducible and reversible cell-specific targeting of AMPARs.

In OPCs and immature OLs, glutamate application evokes AMPAR currents in culture (Barres et al., 1990; Borges et al., 1994; Patneau et al., 1994; Borges and Kettenmann, 1995; Gallo et al., 1996; Yuan et al., 1998) and *ex vivo* in slices (Berger et al., 1992). Electrical stimulation of the hippocampus also elicited glutamatergic currents in NG2+ OPCs, characterized as small inward currents, which could be abolished by blocking AMPARs, and specifically by blocking Ca^2+^-permeable AMPARs (Bergles et al., 2000). Tetanic stimulation of axons in hippocampal slices led to permanent enhancement of EPSCs in NG2+ cells, mediated by Ca^2+^-permeable AMPARs (Ge et al., 2006). Similarly, in thalamocortical slices, stimulation of the ventrobasal nucleus evoked EPSCs in CNP+ NG2+ cells, which could be inhibited by AMPAR antagonist 6-cyano-7-nitroquinoxaline-2,3-dione (CNQX) (Mangin et al., 2012). Moreover, within the CNS WM, synaptic inputs are detected on OPCs (Kukley et al., 2007; Ziskin et al., 2007; Káradóttir et al., 2008, 2005). These morphologically defined synaptic junctions between unmyelinated axons and OPCs in the rat corpus callosum, observed by immunofluorescence and electron microscopy, appeared to be the sites of neurotransmitter release from unmyelinated axons onto Ca^2+^-permeable AMPARs on postsynaptic-like densities on the OPCs (Kukley et al., 2007; Ziskin et al., 2007). However, investigations in DRG and OPC cocultures instead implicated glutamate and ATP release directly from axon varicosities onto nearby OPC processes at axoglial contact sites that lacked the characteristic features of synapses (Wake et al., 2015). This suggests that the promotion of myelination by axonal transmitter exocytosis is not necessarily restricted to a synaptic structure, although some alternative explanations should also be considered, including differences in spinal cord versus cerebral neuron–glia communication, timing of observation at day 1 upon addition of OPCs rather than waiting for 12 h or more for synapses to form, as well as potential artifacts of the coculture system.

### NMDARs on Myelinating Glia

NMDAR activation was reported on OPCs in voltage-clamp recordings. NMDA application on O2A+ cells, which are progenitors of the OL and astroglial lineages, activated an inward current with properties similar to neuronal NMDA currents, including current amplitudes in the range of 20–100 pA, Mg^2+^-dependent rectification at hyperpolarized potentials, and blockade by MK-801 (Wang et al., 1996). In cerebellar slices, a subset of NG2+ OPCs were found to express voltage-gated Na^+^ and K^+^ channels (Káradóttir et al., 2008). From these cells, action potentials could be recorded, and were prevented by blocking AMPAR- or NMDAR-mediated transmission with NBQX or D(–)-2-amino-5-phosphonovaleric acid (D-AP5), respectively. Similarly, in the auditory brainstem, in a subset of pre-myelinating OLs, glutamate stimulation induced firing of action potentials (Berret et al., 2017). *In vitro*, NMDA application appears to specifically regulate OPC differentiation, rather than survival and proliferation, contributing to increased levels of myelination in cocultures of DRGs with OPCs and leading to remyelination in a rodent model of demyelination (Li et al., 2013; Lundgaard et al., 2013).

The effect of NMDAR activity on OPCs is controversial. NMDA application regulated OPC differentiation, rather than survival and proliferation, contributing to increased levels of myelination in cocultures of DRG with OPCs from the cerebellum (Li et al., 2013) or forebrain (Lundgaard et al., 2013). However, application of NMDAR antagonists AP5 and MK-801 to cerebellar slice cultures did not change OPC proliferation, differentiation, branching or extension of their processes, despite observed effects of TTX and GYKI (Fannon et al., 2015). GluN1 deletion from OPCs *in vivo* also had no detectable effect on OPC morphology, basic membrane properties, differentiation, proliferation, or myelination (De Biase et al., 2011; Guo et al., 2012). This finding should be interpreted with caution, however, as GluN1 deletion did lead to an up-regulation in Ca^2+^-permeable AMPARs (De Biase et al., 2011), perhaps compensating for the loss of NMDARs. Second, these studies only looked at earlier time points (no later than P60) but at 10–12 months, mice with OL-targeted GluN1 knockout exhibited neuroinflammation, neurodegeneration, and corresponding severe neurological phenotypes, including hindlimb clasping, hunchback, and ataxia (Saab et al., 2016), indicating different short-term versus long-term actions of oligodendrocytic NMDARs. In line with this reasoning, blocking NMDARs with MK-801 prevented remyelination in rodent demyelination models, including following a cuprizone diet (Li et al., 2013) and ethidium bromide-induced lesion in the cerebellar peduncle (Lundgaard et al., 2013). Taken together, a plausible explanation is that NMDARs do play a role in the differentiation of OPCs into myelinating OLs but this role may be masked by redundancy in the system during development or the roles of NMDARs on both OPCs and neurons.

NMDARs have also been detected on OLs. Pharmacological evidence suggests subcellular targeting of glutamatergic receptors on OLs, with AMPARs enriched on somata and NMDARs on processes (Salter and Fern, 2005). The presence of NMDARs on OLs, including the subunits GluN1, GluN2A,-B,-C,-D, and GluN3A, have been confirmed by RT-PCR, Western blot analysis, and immunostaining (Salter and Fern, 2005; Micu et al., 2006; Li et al., 2013). OLs showed currents evoked by the application of glutamate or NMDA, with partial Mg^2+^ sensitivity, suggesting the presence of functional NMDARs (Káradóttir et al., 2005) with different subunit compositions as compared to neurons, most likely a mixed GluN1/GluN2C/D/GluN3 combination (Káradóttir et al., 2005; Burzomato et al., 2010). Using an array of pharmacological agents, these studies make a strong case for NMDAR-dependent electrical activity in OLs, especially evident following ischaemia induction.

*Ex vivo* Ca^2+^ imaging showed myelinic Ca^2+^ influx following optic nerve stimulation at 50 Hz or following sucrose application to induce vesicular glutamate release, which could be prevented by blocking NMDARs with the glycine site antagonist 5,7-dichlorokynurenic acid (Micu et al., 2016). Genetic ablation of the GluN2D, GluN3A, or both NMDAR subtypes attenuated most of the myelinic Ca^2+^ influx. However, in cerebellar slices under ischaemic conditions, Ca^2+^-sensitive dye-loaded OLs exhibited no change in Ca^2+^ currents in response to activation or inhibition of NMDARs, despite the presence of spontaneous Ca^2+^ currents (Hamilton et al., 2016). Rather than NMDARs, the authors suggested that Ca^2+^ entry into the OLs may be induced by a reduction in cytoplasmic pH until H^+^-gated TRPA1 channels become activated, but this may be a mechanism specific to ischaemia and may not reflect myelinic Ca^2+^ changes following NMDAR activity under normal conditions. Interestingly, glutamatergic receptors are not only expressed on OL lineage cell somata but also on the myelin sheaths (Figure 1D), with sheaths expressing a unique NMDAR consisting of the GluN1 subunit with GluN3, but lacking glutamate-binding GluN2 subunits (Piña-Crespo et al., 2010). This subunit combination renders this myelinic NMDAR insensitive to glutamate, while permitting Ca^2+^ influx into the axon upon activation with glycine or D-serine. Live Ca^2+^ imaging of the optic nerve revealed that this novel receptor had lower Ca^2+^ permeability than other NMDAR subtypes, which may be helpful in preventing Ca^2+^ overload within the relatively small space of the myelinated regions around axons while enabling Ca^2+^ activation even in the absence of glutamate.

### NMDAR Involvement in Myelin Plasticity

Just as potentiation at a classical neuronal synapse enables synaptic plasticity in learning and memory, a mechanism involving axonal activity and NMDAR activation may underlie myelin plasticity. Furthering their observations of decreased myelination following blocked vesicular release, Wake et al. (2011) saw that local MBP translation in response to 10 Hz stimulation was suppressed when axons were pretreated with botulinum toxin or stimulated in the presence of the NMDAR antagonist AP5, whereas AMPAR blockade did not prevent MBP translation. Taken together, these findings suggest that axonal activity may trigger a signaling cascade that acts to target MBP to discrete regions of OPC processes, thus enabling formation and wrapping of myelin around the axon in that discrete region.

The breadth of observations about neuron–glia communication and myelin suggest that myelination may be influenced or regulated by a number of mechanisms. For example, Lundgaard et al. (2013) suggest that neuregulin acts as a myelin “switch”: TTX and MK-801 reduced myelination if exogenous neuregulin was applied, but otherwise showed no effect. Hence, they proposed that neuregulin expression may switch myelination between an activity-independent mode and a NMDAR-sensitive, activity-dependent mode. The latter neuregulin mode is also a faster mode of myelination, which is perhaps why it developed in the CNS to quickly respond to changes and is more prominent during particular developmental time points. Interestingly, neuregulin-1 signaling with its receptor, ErbB, has been previously implicated in myelination: increased type III neuregulin-1 signaling led to thicker myelin sheaths in mice, although normal myelin assembly was observed in conditional NRG1 null mutants until adulthood, and in ErbB3/4 double null mutants until P11 (Brinkmann et al., 2008). Additionally, in conditional ErbB3 mutants, tamoxifen-induced loss of ErbB3 later in life at P19 led to hypomyelination in the medial PFC (Makinodan et al., 2012). For a detailed review of neuregulin-ErbB signaling in nervous system function including myelination, see Mei and Nave (2014).

A different kind of switch has also been proposed, emphasizing the role of OLs in metabolic support (Figure 1F). This switch is thought to developmentally regulate the energy consumption by OLs, initially favoring lipid production and myelination, but later providing glycolytic metabolites like lactate to axons to help support energetically-demanding firing of action potentials (Fünfschilling et al., 2011). Compelling evidence comes from transgenic mouse lines engineered to induce loss of mitochondrial cytochrome C oxidase (COX) function specifically in OLs and at different developmental timepoints. COX is the terminal complex, complex IV, of the electron transport chain in the mitochondria, which produces most of the ATP needed to meet cellular energy demands. Since full loss of mitochondrial function in these animals required several weeks, it is not surprising that initial myelin formation remained unaffected. Based on this study, the authors suggested a model involving OLs switching from an earlier mode of initial myelin formation that utilizes mitochondrial-dependent aerobic respiration to a later mode wherein glycolysis is sufficient for OL survival and provides metabolites, such as lactate, for surrounding axons. Thus, reliance on glycolysis does not appear to have a detrimental effect on OL survival or myelin morphology in the CNS, despite severe consequences in the PNS, raising the possibility that mature OLs may normally provide glycolytic metabolites as a source of energy for axons, similar to the astroglial lactate shuttle at synapses. In a later study, OL NMDAR activation increased the trafficking of the glucose transporter 1 (GLUT1) to OL membranes, an increase in cytoplasmic glucose in OLs, and the concentration of lactate in the culture media (Saab et al., 2016). GluN1 knockouts had reduced myelinic GLUT1, thinner myelin and fewer myelinated axons in the optic nerve from P18 to P21 although this difference was no longer seen at P70. Reduced compound action potential profiles and limited recovery of axonal function following oxygen-glucose deprivation were also observed in GluN1 knockout mice from P19–P21. Taken together, these experiments suggest that once myelin has been formed, OLs switch from a mode of aerobic respiration and myelin formation to a glycolysis-dependent mode that provides metabolic support to axons. It is in the latter mode where NMDAR activation may signal to OLs that a particular axon is firing, and this increased energy demand may recruit increased exchange of metabolites from OLs.

While there is converging evidence that myelinating glia engage in glutamatergic signaling, there are many questions that would benefit from further investigation: How do OLs and OPCs respond differently to glutamate, and what implication does this have for their differentiation, proliferation, and migration? What is the temporal and spatial specificity of the communication between neurons and glia, and what regulates this specificity? And finally, what are the precise signaling pathways following glutamate binding at its receptors – are they similar to neuronal pathways or do glia have their own downstream pathways? Understanding these precise mechanisms will allow for more specific investigations of glutamatergic-dependent myelin plasticity, and ultimately reveal targets for preventing demyelination and promoting repair in myelin-related pathologies.

## Does Myelin Plasticity Have a Critical Period?

Developmental plasticity of circuits is often subject to a “critical period,” a particular time in the organism’s lifetime during which the system is highly adaptable, preceding a period of relative stability or a lack of change in the system. Is myelin plasticity subject to a critical period?

Critical periods for myelination have been observed during development. In the zebrafish spinal cord, OLs form new myelin sheaths during a period of just 5 h, and subsequently, sheaths can only be retracted, with no new sheaths being extended (Czopka et al., 2013). In rodents, early social isolation from P21 to P35 leads to deficits in PFC-dependent behavior and PFC changes including simplified OL morphology, reduced MBP and MAG mRNA, and reduced myelin thickness. These myelin-related and behavioral deficits showed equal severity regardless of whether the social isolation lasted from P21 to P35 or until P65, suggesting a critical period ending after the first postnatal month (Makinodan et al., 2012). Furthermore, rats that were socially isolated as adults (4 months old) did not show changes in the myelin content of the corpus callosum, either in the size of the genu or the number of myelinated axons in the splenium at 6 months (Markham et al., 2009). The inability of later social experiences to have an effect that overrides earlier experiences suggests that mice may have a critical period for myelin regulation of social behaviors.

However, not all brain regions necessarily demonstrate critical periods. The effects of TTX administration on OL number fail to persist by about P30 in rats (Barres and Raff, 1993; Demerens et al., 1996). The thinner myelin and fewer myelinated axons in the optic nerve, as compared to controls, observed in GluN1 knockouts at P18–P21, are no longer seen by P70 (Saab et al., 2016). If early activity-dependent changes in myelination can be mitigated with time, it is possible that myelination remains open to ongoing changes in regions like the optic nerve.

There is also evidence that myelin plasticity continues into adulthood, at least in certain domains. In cell fate mapping experiments, Young et al. (2013) measured the rate of oligodendrogenesis in different regions of the CNS, and saw that new OLs are observed at P21, P60, and in 4 months old adult mice, suggesting that adult-born OLs are involved in continued myelin remodeling. However, these adult-born OLs produced more but shorter myelin sheaths than their earlier-born counterparts. In older rats housed in environmentally enriched conditions from 14 months of age and tested on the Morris water maze, there was increased corpus callosum volume, myelinated fiber and sheath volume, and myelinated fiber length, particularly of smaller axons that were less than 1 μm wide (Zhao et al., 2012). The beginning of this review also enumerated several studies of FA changes in healthy human adults, suggesting training-related myelin plasticity in WM. Looking specifically at older adults (mean age of 61 years), 8 weeks of memory training was still able to drive significant FA changes in certain WM association tracts (Engvig et al., 2012). These studies show that learning-induced WM plasticity can continue to occur into late adulthood. Moreover, changes in connectivity do not appear to occur uniformly across the brain. Rather, it has been observed that distinct networks tend to covary with respect to myelin changes with age (Evans, 2013). Interestingly, these network correlations in myelination changes were shown to potentially increase the resilience of covarying networks to damage, using simulations in which network hubs were deleted (Melie-Garcia et al., 2018). Thus, the specific changes in connectivity that occur in the aging brain may confer improved resistance to increasingly probable network damage as individuals age.

The question of critical periods in myelin plasticity is complex, since it appears to vary depending on the developmental age, neurocognitive domain, and neuroanatomical correlate. Further investigation may resolve questions about which brain networks, if any, are under the regulation of critical periods for myelination.

## Conclusion

Myelin plays an important role in the control of timing in functional circuits, but we are only recently beginning to elucidate how it may interact with neuronal activity to facilitate experience-dependent plasticity. Myelin plasticity is a challenging phenomenon to study, as it is difficult to discriminate from neuronal mechanisms, and myelination varies based on developmental age, anatomical region, and neuronal identity. We have reviewed evidence of how neuronal activity alters myelination from gross WM changes to cellular and molecular changes involving glial proliferation, differentiation, morphology, and the features of myelin. This neuron–glia interaction has been shown to be mediated in part through glutamatergic signaling, but it is yet to be determined exactly how this signal may manifest changes in myelination. For example, it may enable axo-glial potentiation driving local myelin synthesis, or it may identify the cells in need of metabolic support. Continued investigations that will elucidate more specific mechanisms, critical periods, and functional outcomes of myelin plasticity will be critical in advancing our understanding of myelinated circuitry in experience-dependent plasticity.

## Author Contributions

This review was written by ZC with feedback and editing by RTK and ESR.

## Conflict of Interest Statement

The authors declare that the research was conducted in the absence of any commercial or financial relationships that could be construed as a potential conflict of interest.
